# A feasibility randomised trial of remotely delivered Video Interaction Guidance for parents of children with intellectual disability referred to specialist mental health services

**DOI:** 10.1186/s40814-026-01800-2

**Published:** 2026-03-11

**Authors:** Vaso Totsika, Eilis Kennedy, Michael Absoud, Rachel McNamara, Elizabeth Randell, Gemma Grant, Angela Casbard, Sophie Levitt, Angela Hassiotis, Manuel De Oliveira Gomes, Cristina Di Santo, Kian Fortes, Charmaine Kohn

**Affiliations:** 1 Division of Psychiatry, University College London, London, UK; 2 Tavistock & Portman NHS Foundation Trust, London, UK; 3 Department of Clinical, Educational and Health Psychology, University College London, London, UK; 4 Evelina London Children’s Hospital, South London and Maudsley NHS Foundation Trust, London, UK; 5 Centre for Trials Research, Cardiff University, Cardiff, UK; 6 Challenging Behaviour Foundation, Chatham, UK; 7 Brighter Futures for Children, Reading, UK

**Keywords:** Intellectual disability, Video Interaction Guidance, Parent, Mental health

## Abstract

**Background:**

Children with intellectual disability are at a higher risk of presenting with behaviours that challenge. Video Interaction Guidance (VIG) is a brief, personalised, strengths-based therapy that focuses on improving the parent–child relationship and interactions. A strong parent–child relationship may reduce the risk of behaviours that challenge. Access to support is difficult for families of children with intellectual disability. Remotely delivered support may enhance access to therapy for those who might otherwise struggle to access support. To date, no definitive effectiveness trial of VIG, or remotely delivered VIG, has been conducted, including in intellectual disability.

**Methods:**

A feasibility randomised controlled trial (RCT) aimed to recruit 50 parents of a child with intellectual disability (aged 6–12) referred to specialist mental health services. Participants were randomised on a 1:1 basis to VIG plus treatment as usual (TAU) or just TAU. Measures were collected remotely at baseline, 3-month, and 6-month follow-up. A parallel economic study explored the feasibility of a future economic evaluation, while an embedded process evaluation explored feasibility and acceptability through qualitative interviews. A survey investigated TAU in specialist mental health services. A Parent Carer Advisory Group of 10 parents of children with intellectual disability worked with the research team on design, recruitment, data analysis and interpretation.

**Results:**

Forty-four parents consented to participate in the study and 40 were randomised to the RCT. Of those, 75% remained in the study at 6-month follow-up, and 70% of the VIG-arm participants completed at least 3 cycles of VIG. At 6-month follow-up, between 83.3% and 100% of parent-completed questionnaires were completed, including the Developmental Behaviour Checklist (DBC2 at 86.7% completion rate). The acceptability of VIG was high among parents and practitioners. Parents identified few barriers to participation when VIG was delivered remotely. The cost of VIG was calculated at £153.35 per session and £306.70 per cycle. Video-feedback interventions are not typically part of TAU: just 15% of 66 specialist mental health services reported offering any video-feedback intervention to parents of children with intellectual disability.

**Conclusions:**

Findings supported the feasibility and acceptability of a definitive trial of remotely delivered VIG to parents of children with intellectual disability referred for support to specialist mental health services. Adaptations will be needed to enhance recruitment and align some of the study outcomes and processes more closely to parent preferences.

**Trials registration:**

ISCTRN 13171328, Registration date 28 December 2022.

**Supplementary Information:**

The online version contains supplementary material available at 10.1186/s40814-026-01800-2.

## Key messages regarding feasibility

- Video Interaction Guidance (VIG) comes under the umbrella of video-feedback interventions which are considered effective for improving parent–child relationship quality, but VIG’s effectiveness with parents of children with an intellectual disability has not been investigated to date. The lack of any research on VIG with these families indicated a need to test feasibility and acceptability of the intervention and its evaluation prior to an effectiveness study.
- The study demonstrated that it is possible to recruit parents whose children with an intellectual disability have been referred to specialist mental health settings and that parents remain in the study at 6 months. The study demonstrated that it is possible to offer remotely at least 3 cycles of the intervention to these parents. VIG was acceptable to parents and clinicians, and parents found the remote delivery mostly highly acceptable.
- This study successfully met its progression criteria, suggesting progression to an effectiveness study would be warranted. Changes in the age of the children and the intervention delivery sites are the main changes needed to improve recruitment in the main study, while participants, clinicians and the Parent Carer Advisory group were strongly in favour of a primary outcome that measures parent–child relationship.

## Background

Intellectual disability is a neurodevelopmental condition that is characterised by aetiologically diverse challenges in cognitive functioning (typically identified as an IQ below 70) and adaptive skills (skills needed for daily functioning, e.g. communication, personal care, social skills), evident in the developmental period [1]. Between 30 and 50% of children with intellectual disability experience a diagnosable mental health problem, often presenting with behaviours that challenge [2]. These challenges place a significant burden on families who need support to manage effectively at home. Where families feel unable to manage these behaviours, children often end up in residential schools away from home [3]. In England, support is provided by specialist mental health services, where children with suspected mental health problems and/or behaviours that challenge are referred to by primary care, education or social care. Such services are standalone services or integrated within neurodevelopmental services or within all-age intellectual disability services. Services are under strain and referred children typically do not access support in a timely manner, with many waiting over 2 years [4]. Whilst families are waiting for support, they get very little contact or input from specialist mental health services [5]. Families themselves indicate high levels of unmet need for support from services and very low levels of intervention receipt [6]. Digitally enabled care, including interventions and support offered remotely, had been proposed as a way to improve access to mental health support for families and children with an intellectual disability [7]. 

Research into the developmental pathways of behaviours that challenge and emerging mental health problems in intellectual disability points to the role of the parent–child relationship as a risk factor for the development of such difficulties [8–10] and, also, as the mechanism that carries over the risk stemming from other adversities (e.g. poverty, maternal mental health problems [11]). UK population data indicate that in families with a child with an intellectual disability, there are higher levels of conflict and lower levels of closeness in the parent–child relationship compared to families with a child without intellectual disability [10]. Evidence, however, points to the potential to significantly improve the quality of parent–child relationships through intervention and support to these families with associated gains for child developmental outcomes [12].

Video Interaction Guidance (VIG) is an intervention designed to enhance parent–child relationship quality through its focus on attunement during parent–child interaction episodes [13]. Based on the developmental theory of intersubjectivity [14], VIG focuses on moments of successful interaction and communication between two partners to identify strengths in their relationship and promote a more positive affective relationship [13]. It uses video as a therapeutic tool where the parent sees themselves on video and is guided by the VIG practitioner to identify what they are already doing well in the way they interact and communicate with their child. VIG is expected to have a positive impact on child behaviours that challenge through the building of a more positive relationship between the parent and the child [15]. Therefore, VIG could potentially be a helpful intervention for parents of children with an intellectual disability who present with behaviours that challenge or mental health problems. Video-feedback interventions (not VIG specifically) are recommended by NICE (National Institute for Health and Social Care) for parents of adopted children (NG26 [16]), autistic children (CG170 [17]) and very young children (PH40 [18]) where there is a need to strengthen communication and parent–child interaction. No effectiveness trial of VIG has been conducted with any group of children; to date, only two small studies of VIG have been conducted: a small pilot trial (*N* = 31) with preterm neonates [19], and a small (*N* = 19), uncontrolled feasibility study with infants [20].

VIG, which was offered remotely during the COVID-19 pandemic, could be part of the digitally enabled care provided by specialist mental health services to support families more efficiently. VIG maintains its key therapeutic mechanisms when VIG practitioners support a parent remotely (for example, over Teams or Zoom) compared to face-to-face guidance [21].

### Aims

The present study was a feasibility randomised controlled trial of remotely delivered VIG aiming to examine (1) the recruitment rate of parents of 6–12 year-old children with an intellectual disability referred to specialist mental health services, (2) participant retention in the study 6 months after randomisation and (3) completion of at least 3 cycles of VIG (1 cycle includes two meetings with a VIG practitioner about a fortnight apart). Secondary questions examined (1) the completeness of parent-reported outcome measures at 6 months follow-up, (2) the acceptability of VIG by parents and practitioners, (3) the feasibility of remote implementation of VIG in terms of perceived effectiveness, likely adaptations and any unintended implementation failures and (4) the feasibility of collecting data on wider service use and the preliminary estimation of costs of VIG as a means of investigating the feasibility of a future health economics study.

### Study design

This was a feasibility randomised controlled trial, with participants randomised on a 1:1 allocation basis to receive VIG plus treatment as usual (TAU) offered by their specialist mental health service or just TAU. Full methods are detailed in the study protocol [22], a brief description follows. Parent-reported measures were collected through an online survey prior to randomisation (baseline), and 3 and 6 months after randomisation. An embedded process evaluation included qualitative interviews with clinical staff and parents from both arms. Study participants received a small monetary ‘Thank you’ in the form of a gift voucher following completion of each survey and qualitative interview. A parallel feasibility economics evaluation drew data from a telephone interview with participants at baseline and 6 months post-randomisation as well as an online survey with practitioner-reported information at 3 months post-randomisation. A UK wide survey aimed to capture information on interventions and supports offered by specialist mental health services as part of TAU. Part of the TAU survey has already been published [5] but data related to the use of video-feedback interventions and VIG are reported here. No changes were made to method following publication of the protocol and the analysis followed pre-specified progression criteria. Ethical approval was provided by London-South East Research Ethics Committee (IRAS Number 315829). A 10-member Parent Carer Advisory Group (PCAG) guided every stage of the study, including this publication (see Table 1).

**Table 1:** Patient and Participant Involvement and Engagement (PPIE) in the VIG-LD trial reported according to GRIPP2-SF^1^ [43]

GRIPP2-SF areas	Description
Aim	The aim of the 10-member PCAG was to ensure the study was informed by the lived experiences of parents and carers of children with intellectual disability. PPIE sought to enhance the acceptability of the intervention, refine the research methodology, ensure that the study captured outcomes meaningful to families and support dissemination
Methods	Direct editing of materials was co-ordinated over email PCAG members participated in a study recruitment video and a finding dissemination video. PCAG members co-produced the content of the video, also guiding selection of background, sound and images Two PCAG members collaborated in writing the TAU survey and the current paper. All PPIE input was co-ordinated by the PPIE lead, the Challenging Behaviour Foundation
Study results	1. The language used in participant-facing documents 2. Guided the analysis of TAU survey results and directly analysed qualitative data 3. One video recruiting families to the study, which likely contributed to the increased participant diversity achieved 4. RCT and Process evaluation analyses: valuable insights into the real-world impact of the intervention. PCAG added context around some of the process evaluation findings 5. Contributed to the conclusions drawn, ensuring that the outcomes reflected families’ lived experiences and priorities 6. A second video to disseminate findings 7. Significant contribution to design of amendments for final trial (see Discussion)
Discussion and conclusions	There was a high level of commitment by PCAG members who were incredibly quick to respond to opportunities to participate in study activities The PCAG group’s contributions to recruitment, particularly through the recruitment video, demonstrated the power of co-designed resources. Hearing directly from parents helped build trust with potential participants and was a key factor in overcoming recruitment challenges. The PCAG’s input was critical for the interpretation of findings and for identifying changes needed for the future trial
Reflective/critical perspective	Family carers were positive about being involved in the study but were also able to be a critical friend to the research team and provide constructive feedback and fresh ideas The study took place over 3 years and many months passed between meetings which could disrupt communication and sense of involvement. Whilst some email updates were shared in between meetings these were limited by resource and capacity constraints. The PCAG recommended bimonthly written updates and meetings with a minimum duration of 2 h to allow a full recap of information on each occasion. Nevertheless, the PCAG remained committed to the study despite PCAG members juggling multiple responsibilities e.g. caring commitments, employment

^1^*GRIPP2-SF* Guidance for Reporting Involvement of Patients and the Public Checklist Short Form, *PCAG* Parent Carer Advisory Group

### Participants and settings

Participants were included if (1) they had a minimum age of 18 years, (2) were a parent (biological, adoptive, foster, step mother or father, or any other caregiver), (3) had a child aged between 6 and 12 years old, (4) the child’s intellectual disability was identified by a clinical diagnosis of intellectual disability or eligibility to receive intellectual disability services; eligible children could have any associated neurodevelopmental conditions (e.g. autism) or other co-occurring problems; (5) the child achieved a composite score of < 80 on Vineland Adaptive Behaviour Scales (VABS 3 [23]) which demonstrates limitations in adaptive skills consistent with intellectual disability and (6) the child has been referred to a specialist child mental health service in England.

Specialist mental health services for this population vary widely in name and setup [24]. In our study, settings were identified if they provided support for mental health problems or behaviours that challenge children with an intellectual disability as part of National Health Service (NHS) services that children can access following referral from primary care, schools, or other services. Specialist mental health services can be Child and Adolescent Mental Health Services (CAMHS) specific for this group of children (e.g. Learning Disability CAMHS; LD CAMHS) or CAMHS that see children with neurodevelopmental conditions. Services can also be integrated within paediatric neurodevelopmental services with a pathway for child behaviour or mental health problems or within all-age intellectual disability services with a pathway for children’s mental health or behaviours that challenge. We aimed to recruit 5–7 sites throughout England.

Potentially eligible participants were initially provided with information about the study from the clinicians. If they were interested, parents contacted the research team to express an interest in the study. At that stage, the researcher made an appointment for a telephone meeting to go through the Participant Information Sheet and answer any questions the parent may have had. A consent form was sent following the meeting, and if the parent consented, they were recruited into the study. An appointment was then made to undergo formal screening to determine eligibility. Exclusion criteria were (1) to already participate in the trial for another child, (2) to live with the target child < 50% of the time (e.g. if the child is in a residential school or an inpatient unit), (3) to be in receipt of a similar video-feedback intervention on the day of the screening (Video feedback Intervention to promote Positive Parenting: VIPP; VIPP-Sensitive Discipline: VIPP-SD; Marte Meo, Video Parent–Child Interaction: VPCI, Paediatric Autism Communication Therapy: PACT) either remotely or in person, and (4) the family to be under active family court proceedings. The present paper followed CONSORT guidance for reporting (see Supplementary material 1 file for the CONSORT checklist).

### Intervention

VIG is a brief, strengths-based model that focuses on parent–child successful communication and interaction and uses video as a therapeutic tool. Following an introductory session, a trained VIG practitioner meets the parent to videorecord a successful exchange between the parent and the child. The practitioner edits the video to identify the most attuned moments of interaction between the parent and the child. A second meeting follows where the parent watches the edited video clips whilst being guided by the practitioner to identify those successful moments of interaction with their child. This second meeting is called a shared review. The two meetings constitute 1 cycle of VIG. Three cycles are typically offered, though given the complexity of child–parent needs, parents or practitioners could request to extend up to a total of five cycles in the present study. Meetings took place over Microsoft Teams, though in-person sessions were allowed if deemed preferable by parents or practitioners. At the start and end of the cycles, parents are guided to identify and score up to three goals (Goal Based Outcomes; GBO [25]). For the purposes of the study, it was anticipated that the intervention would have been completed over a 12-week period.

Practitioners were trained according to the specifications of AVIGuk, the association for Video Interaction Guidance in the UK: for practitioners with no prior training, a 2-day (online or face-to-face) training session with an accredited trainer and supervisor took place (the training can take place over the course of 4 half-day sessions as well). Following completion of the training, trainee practitioners start delivering VIG whilst being supervised (for every cycle) by an accredited VIG supervisor. VIG practitioners were regular staff of specialist mental health services participating in the study (e.g. psychologists, speech and language therapists, ABA therapists, mental health practitioners and others).

### Outcomes

Outcomes were pre-defined in a statistical analysis plan and a qualitative analysis plan. To address the three primary feasibility questions, outcomes were (1) the recruitment rate defined as the proportion of participants randomised among those expressing an interest in the study, (2) the retention rate defined as the proportion of participants with at least one parent-reported questionnaire completed at 6-month follow-up among those randomised and (3) the proportion of participants who completed 3 cycles of VIG (of a maximum 5) among all participants randomised to the VIG arm. Additional planned outcomes (related to the primary questions of recruitment and retention) were (1) the estimation of the consent rate (the proportion of those who consented among all those who expressed an interest in the study); (2) the eligibility rate (the proportion of consenting families deemed eligible following screening among those expressing an interest); and (3) the proportion of participants who at 6-month follow-up completed the Developmental Behaviour Checklist-2 (DBC-2 [26]) among those randomised. The DBC-2 is a measure of behavioural and emotional problems in young people with intellectual disability [26]. To address the secondary feasibility questions, outcomes were (1) the proportion of participants who provided useable data on each parent-reported questionnaire measure, estimated separately at each time point; (2) the acceptability of VIG to parents and clinicians estimated by the data on the qualitative interviews after the 6-month follow-up; (3) the feasibility of remote implementation of VIG, estimated by the qualitative interviews, quantitative data on VIG fidelity, and quantitative data on patient satisfaction with clinical services; (4) the feasibility of capturing health economic (HE) data in relation to the use of services by the parent and the child (including medication use) through participant phone interviews and the feasibility of costing VIG and TAU. Additional outcomes (related to the secondary objectives) included (1) descriptive statistics on parent-reported outcomes across study time points; (2) a comprehensive description of Treatment As Usual (TAU) interventions offered and received, defined as (a) the proportion of specialist mental health services (from the UK-wide survey) offering video-feedback intervention altogether and by stage of a family’s referral stage (waiting list, assessment, treatment stage), (b) the proportion of participants in the TAU arm who received any video-feedback intervention (VIG, VIPP, VIPP-SD, Marte Meo, VPCI, PACT) as reported by participants at the 3-month follow-up; (3) the acceptability of the trial design, study methods, delivery and future adaptations proposed by parents and clinical staff in the qualitative interviews that took place shortly after the 6-month follow-up.

We used a traffic light system [27] to prespecify progression criteria for the first primary feasibility objectives. A green signal for recruitment rate was defined as recruiting > 50% of interested parents, 35–50% amber, and < 35% red. A green signal for study retention rate would be to retain > 70% of participants at 6 month follow-up (60–69% amber, < 60% red). A green signal for VIG completion would be > 80% completing 3 VIG cycles (65–79% amber; < 65% red). These three primary feasibility outcomes were deemed the basis for determining whether progression to a full trial was possible. Beyond these, we also used a traffic light system to inform decisions about the measures to be used in a final trial: a green signal for measure completeness was defined as 100–80% of useable data for DBC-2 scores (likely primary outcome in full trial) at 6-month follow-up. Any measure with < 70% useable data at 6-month follow-up would be reconsidered.

### Sample size

Information from similar studies [20, 28] was used to inform the (green) target recruitment rate of 50%. We used a hypothesis testing approach to determine the feasibility of recruitment [29] as this was the primary feasibility objective. With the lower limit of the green zone set to 50% and the upper limit of the red zone set at 35%, then with 90% power and 5% one-sided alpha, we needed to approach and invite 97 families. The threshold value was 42: an observed estimate below this cut-point would mean a non-significant result (*p *= 0.05) and a value at or above the cut-point would be significant (*p* < 0.05). Thus, if recruitment was within the amber zone but was at least 42, then minor changes would be required to improve recruitment; if recruitment was < 42, then major changes would be required.

### Randomisation

Participants were randomised on a 1:1 basis to the two study arms (VIG + TAU vs TAU only). The randomisation list was produced by a statistician using random permuted block randomisation stratified by site. Randomisation took place after participants had consented to the study and had their eligibility confirmed through the screening interview with a researcher. Randomisation was performed by the Senior Trial Manager who was independent from data collecting researchers. The Senior Trial Manager notified the clinical site and the Principal Investigator of each participant’s group allocation. Researchers collecting data from participants remained blind to group allocation until after completion of the 6-month follow-up; data collection to this point was via online survey where participants self-identified their group allocation within the survey software. Researchers remained blind to group allocation up to the point where qualitative interviews were undertaken with participants who had given separate prior consent to be interviewed. Qualitative interviews were conducted on a rolling basis and only after each participant had completed their own 6-month follow-up survey. This sequencing was used to avoid any potential influence of qualitative interviews on quantitative outcome responses.

### Approach to analysis

Descriptive statistics were used to analyse the outcomes related to the primary feasibility objectives (recruitment, retention and VIG completion). For recruitment, a binomial probability test examined if the recruitment rate achieved was equal to or smaller than 35%, which was set as the upper limit of the red zone. Descriptive statistics were used to analyse quantitative outcomes related to secondary feasibility objectives, i.e. measure completeness, VIG fidelity, patient satisfaction with clinical services, HE data capture and intervention cost estimation. Qualitative analyses were guided by the Medical Research Council (MRC) framework for process evaluation [30], with a focus on implementation. Different analytic approaches were used to reflect the specific aims of each qualitative component. For the analysis of intervention acceptability, Framework Analysis guided by the Theoretical Framework of Acceptability (TFA) [31] was used as acceptability was a pre-defined construct with clearly defined domains. This allowed for systematic mapping of parent and clinician accounts to the seven acceptability domains and comparison across participant groups. For all other qualitative components not directly related to intervention acceptability (including barriers and facilitators to remote delivery, perceived effectiveness, adaptations, and experiences of the trial), reflexive thematic analysis [32] was used to allow inductive exploration of participants’ accounts. Qualitative analysis was led by an experienced senior qualitative researcher, with contributions from research assistants and discussion within the wider research team. Analytic decisions and interpretations were reviewed and refined through regular team meetings and validity-focused discussions, with senior qualitative oversight. PPI input further informed interpretation of findings. NVivo software was used to support data management and analysis.

## Results

### Primary feasibility outcomes

#### Participant recruitment

The study recruited from five NHS Trusts across England (two in London, one in East of England and two in North West England). Recruitment was open between February 2023 and February 2024. In total, 403 families were provided with study information, distributed via clinicians (*n *= 154) and clinical site mailouts (*n *= 249). Recruitment efforts included approaching families from referral lists (*n* = 3), waiting lists (*n *= 111), active caseloads (*n *= 207), and an unknown origin where screening was completed by local Research and Development (R&D) teams (*n* = 82). From these efforts, the study team received 107 Expressions of Interest (EOIs), with 296 families not responding. Among those who expressed interest, 44 provided consent. Of the 63 who did not consent, reasons included ineligibility (*n* = 1), active decline (*n* = 19), and no response or assumed withdrawal of EOI (*n* = 43). Eligibility screening determined that of the 44 consenting participants, 1 was ineligible. A further participant later declined and 2 failed to respond, leaving 40 consenting participants screened eligible to proceed to randomisation. Randomisation was done on a 1:1 basis, so of the 40 participants randomised, half (20) were allocated to the VIG + TAU group, while 20 were allocated to the TAU group. All recruitment rates estimated below are based on the total number of participants recruited and randomised in the study.

**Fig. 1:**
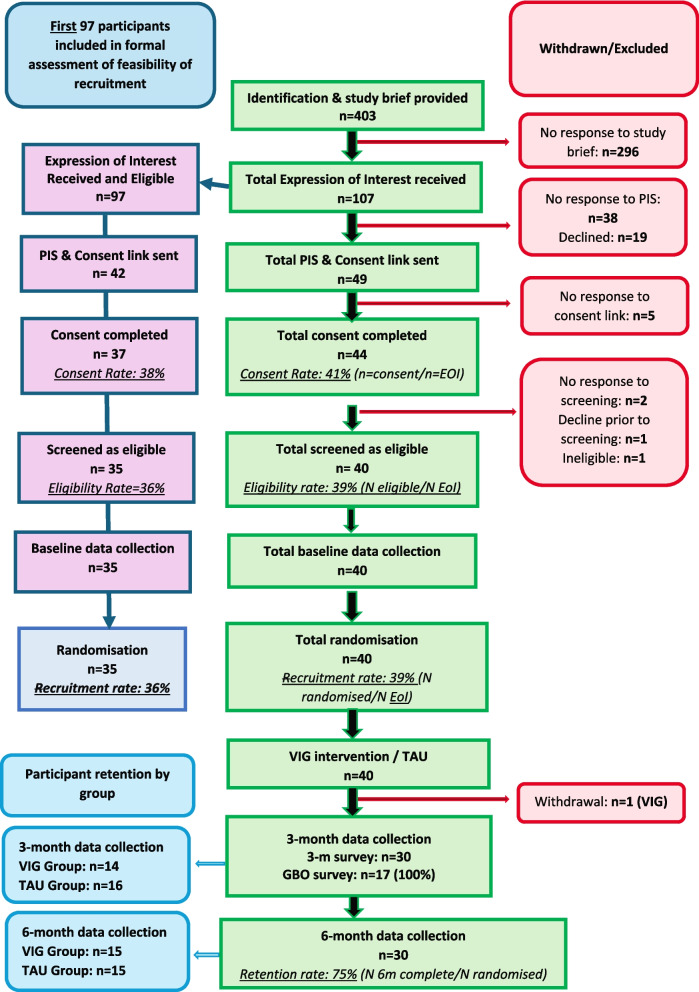
CONSORT flowchart of participant recruitment in the VIG-LD trial

Figure 1 indicates the flow of participants in the study. The recruitment rate was estimated using data from the first 97 parents to express an interest in the study: the recruitment rate was 36% (95% CI 27–46%). The one-sided binomial test gave a *p*-value of 0.44, meaning we were not able to reject the null hypothesis that the true recruitment rate was equal to or less than 35%. A sensitivity analysis estimating the recruitment rate based on the last 97 EOIs received found a similar recruitment rate of 37% (see Table 2). The recruitment rate fell within the amber zone, with 35 participants randomised from the first 97 EOIs. As the recruitment numbers fell below the critical value of 42, this criterion highlighted the need for major changes to improve recruitment in a future trial. Tables 3 and 4 include demographic information on participants and their child with intellectual disability.

**Table 2:** Recruitment activity in the trial

	Based on *all* 107 EOI	Based on *first* 97 EOI (traffic light criterion)	Based on *last* 97 EOI (sensitivity analysis)
Consent			
N consented participants	44	37	44
Consent rate (*n* = consented/*n* = EOI)	41%	38%	40%
Eligibility screening			
N screened eligible participants	40	35	36
Eligibility rate (*n* = eligible/*n* = EOI)	39%	36%	37%
Randomisation			
N of randomised participants	40	35	36
Recruitment rate (*n* = randomised/*n* = EOI)	39%	36%^1^	37%

^1^This is the pre-determined primary feasibility criterion

**Table 3:** Demographic characteristics of parents of children with intellectual disability randomised to the trial

	*N* = 40
Participant male gender	9 (22.5%)
Participant living with partner/spouse	32 (80.0%)
Participant single	8 (20.0%)
Participant ethnicity Asian Black White Other Not stated	4 (10.0%) 3 (7.5%) 29 (72.5%) 3 (7.5%) 1 (2.5%)
Participant in paid employment	15 (37.5%)
Other adult in household in paid employment	27 (69.2%)
Participant educated to degree level	17 (42.5%)
Family struggling financially	20 (50.0%)
Family experiencing difficulties with paying bills	25 (62.5%)
Household is in relative income poverty (OECD^1^-equivalised)	24 (60.0%)
N of adults in HHD median (IRR^2^, min–max)	2 (0.00, 1–4)
N of children younger than 13 years median (IRR, min–max)	2 (1.75, 1–5)
N of children older than 14 years median (IRR, min–max)	0 (1.00, 0–8)

^1^*OECD* Organisation for Economic Co-operation and Development

^2^*IRR* inter-quartile range

**Table 4:** Characteristics of children with intellectual disability for the 40 randomised parents

	*N* (%)
Child male gender	34 (85.0%)
Child living with participant full-time	40 (100.0%)
Child intellectual disability diagnosis (or suspected^1^)	23 (58.5%)
Child (global) developmental delay diagnosis (or suspected)	17 (42.5%)
Child autism diagnosis (or suspected)	36 (87.8%)
Child communication disorder (or suspected)	16 (39.0%)
Child has motor disorder (or suspected)	6 (14.6%)
Child has ADHD (or suspected)	25 (61.0%)
Child has other genetic syndrome (ADNP, CdL, FOXP1)	5 (12.2%)
Child has EHC^1^ plan	37 (92.5%)
Child is currently assessed for a EHC^2^ plan	3 (7.5%)
Child is registered with a school	39 (97.5%)
Child is educated in mainstream school and classroom	5 (12.8%)
Child is educated in specialist unit, classroom or school	34 (87.2%)
Child age median (IRR, min–max)	9 (2, 6–12)
Child Adaptive Behaviour Composite (VABS 3) mean (SD, min–max)	46 (14.78, 22–70)

^1^Participants were invited to indicate any diagnosis of neurodevelopmental condition their child had been given or was currently being assessed for or awaiting assessment

^2^*EHC* Educational, Health and Care plan is the statutory document in the English education system recognising a child has special educational needs or disabilities

Recruitment was undertaken by the research team with support from local R&D teams. Several factors significantly impacted recruitment, with only two research sites achieving the tentative recruitment target of 10 participants (one in East of England consented 10 participants and one in North West consented 15 participants). The remaining research sites achieved a recruitment total of six (North West), seven (London), and six (London). Challenges included considerable delays in local NHS Trust approvals during site setup and overestimation of eligible families by some sites. Recruitment was better in sites where clinical or R&D teams within the site had capacity to follow up with telephone calls to potential participants whom they had sent information about the study.

#### Participant retention

At 6-month follow-up, 30 parents took part in the survey. Of those, all 30 had completed at least one measure. The retention rate was thus estimated as 75% (Fig. 1), placing this primary feasibility objective within the green zone of feasibility. The retention rate was the same across both arms (Fig. 1). Retention strategies included regular contact with families, reminders for follow-up appointments, and the flexibility of remote data collection.

Parents reported that their continued participation was motivated by the perceived relevance of the intervention and their desire to improve support for themselves and their families. Retention to the study was supported by the fact that questionnaires were completed online so it was easy for participants to access and complete them at a time that was suitable for them. However, some families reported challenges with maintaining engagement during the follow-up period, particularly when managing multiple competing priorities.

#### Intervention adherence

VIG adherence was defined as the completion of three VIG cycles, the aim set by the Association for Video Interaction Guidance in the UK (AVIGuk). Fourteen of the 20 VIG arm participants had completed at least three cycles of VIG, an adherence rate of 70%. This places the objective within the amber zone of feasibility. Barriers to adherence included scheduling conflicts, technological difficulties with remote participation, and the reflective nature of the shared review sessions, which one parent found challenging. A deviation from the pre-specified analysis plan was with regard to the data source to be used to analyse this objective: to estimate adherence, we initially planned to analyse participant self-reported data at the 3-month follow-up. The 3-month follow-up coincided with the anticipated completion of VIG at the expected 12-week intervention period. It was not possible to estimate adherence from these data because (1) not all 20 VIG participants took part in the 3-month follow-up; (2) participants were not all aware which group they had been allocated. Of the 30 participants at the 3-month follow-up, 17 self-reported as being in the VIG group. Of those, only 13 had been randomised to the VIG group. For these reasons, adherence was calculated from VIG supervisor data.

### Secondary feasibility outcomes

#### Completeness of parent-reported measures

Completeness of outcome measures was defined as the proportion of participants who provided useable data on each parent-reported measure, estimated separately at each time point. Measured completeness ranged between 100% and 97.5% at baseline, 90% and 86.7% at 3-month follow-up and 100% to 83.3% at 6-month follow-up. Further, 86.7% completed the DBC-2 at 6-month follow-up. Findings suggest none of the parent-reported measures was eligible for removal from the final trial.

#### Acceptability, barriers, and facilitators of offering/engaging with VIG

We interviewed 10 parents and 9 staff as part of the process evaluation. The acceptability analysis found remotely delivered VIG to be highly acceptable to parents (“I love it… I really enjoyed it” P5) with minimal burden and no opportunity costs reported. Parents viewed VIG as a positive and impactful intervention, appreciating its ability to enhance their awareness of their child’s communication skills, improve parental confidence by affirming their positive and effective behaviours (“It’s rewarding to know what you’re doing right.” P5) while reducing self-doubt and guilt (“I learned a lot about myself as a parent that I shouldn’t be hard on myself” P5; [VIG] “does help you to be reflective without being worried about being judged which I think is really good” P4; “It alleviated some of that mum guilt” P3). For parents, barriers to engaging with VIG remotely included challenges with the child’s readiness to participate and difficulties with self-reflection during video reviews. However, the flexibility of scheduling, the convenience of home-based delivery, and reduced travel requirements were seen as significant facilitators of this delivery format. Parents particularly valued the minimal intrusion of remote delivery, integrating easily into daily routines, enabling greater accessibility and engagement.

Clinicians found VIG to be highly acceptable, emphasising its transformative and powerful impact on parent–child relationships (“It feels enormously transformative in their day-to-day lives.” S1; “Without VIG I would never have been able to have convinced mum that she did have a lot of gifts and so did her daughter. It was just such a beautiful thing to do with them” S8) and its strong alignment with professional values (“How can you not love an intervention that puts the parent and child at the centre?” S2). They described the intervention as highly positive, fostering meaningful interactions and empowering parents, with minimal ongoing burden and low resource demands. Despite some resource and remote delivery challenges, clinicians expressed confidence in VIG’s effectiveness and adaptability, viewing it as an impactful and invaluable addition to their therapeutic work. Barriers to remote delivery identified by clinicians were technological issues, parent and child capacity for engagement and their own preference for hybrid delivery. However, parent preference for remote delivery reduced travel and resource demands on their service and the effectiveness of this format in capturing naturalistic parent–child interactions was identified by staff as important facilitators. Clinicians noted that remote VIG increased accessibility for families and allowed for flexible, efficient clinical workflows, supporting its continued use in appropriate contexts. Supplementary material 1: Tables S1–S4 include the full acceptability framework by parents and clinicians as well as barriers and facilitators identified by each group [see Supplementary material 1].

#### Feasibility of intervention, perceived effectiveness, likely adaptations and unintended implementation failures

VIG was identified as a highly adaptable intervention suitable for a wide range of families, including those with children who have varying levels of intellectual disabilities, neurodivergent parents, or parents with low confidence. Its emphasis on capturing non-verbal communication and fostering parent–child relationships was seen as making it particularly suitable for families with unique communication needs. Staff highlighted VIG’s ability to complement existing services by focusing on relational dynamics, offering a supportive and strengths-based approach that enhances parent confidence and skill-building.

In terms of implementation within the clinical pathway, staff viewed VIG as valuable for building rapport with the family, supporting initial formulations, and complementing or consolidating other treatments. Staff suggested VIG’s flexibility allows it to be used as a standalone intervention or alongside other therapies, depending on family needs. Adaptations made during the trial included extending timelines due to family scheduling conflicts, accommodating alternative filming methods, and adapting the final shared review process to meet individual needs.

No unintended implementation failures were reported. Staff expressed strong enthusiasm for continuing VIG. However, resource constraints, such as staff shortages or lack of experienced senior clinicians, were noted as challenges to scaling its delivery. To address this, staff and managers emphasised the value in secure funding, ongoing promotion within clinical networks, and developing long-term integration plans to embed VIG as part of treatment as usual.

We measured satisfaction with the clinical service that participants received using the 9 Experience of Service Questionnaire (ESQ) Satisfaction with Care scale [33]. At 3-month follow-up participants in the TAU group reported significantly higher levels of satisfaction compared to participants in the intervention arm (TAU mean EPS = 21.21, SD = 5.82, VIG mean = 16.54, SD = 4.70; Hedges *g* = − 0.85, 95%CIs − 1.62, − 0.0.77).

We examined the fidelity of VIG delivery by estimating the percentage of shared reviews receiving a score that corresponds to the VIG practitioners’ level of accreditation and training [34]. We included 13 randomly selected shared reviews (balanced across 1 st, 2nd, and 3rd VIG cycles) across different practitioners and sites and found that all sessions were delivered with 100% fidelity. Practitioners were mostly (12/13) at the initial stages of training.

Service use and medication data were collected during an interview with the parent using a modified version of the Child and Adolescent Service Use Schedule (CA-SUS) [35]. Interviews were conducted at baseline (*N* = 33) and at 6 months post-randomisation (*N* = 22), with a retention rate of 66.67%. Attrition rates were higher for TAU participants (42.11%) compared to VIG-LD (21.43%). Item-level missing data were minimal (< 1%) and were mainly due to recall difficulties. Qualitative feedback from the interviewers highlighted that the main difficulty in capturing CA-SUS data was arranging phone interview appointments, particularly around the 6-month mark. While most participants found the structured interview straightforward and acceptable, there were some reports of communication challenges due to language barriers (e.g. one interviewer indicated they had difficulty understanding participants over the telephone because of different accents, and some participants did not quite understand the interviewer), as well as the survey taking more time than expected, especially when discussing service use and medication details. Future research should consider a hybrid data collection approach that integrates structured online surveys with brief follow-up calls to improve data completeness and participant engagement.

The cost of VIG-LD was estimated based on the salary bands of practitioners, using unit costs from the Personal Social Services Research Unit (PSSRU) [36]. Salary bands refer to the pay scale or salary level of the staff within the NHS Agenda for Change (AfC) pay system [37]. These bands determine the hourly wage or salary of the healthcare professional, which was then used to estimate the cost of VIG-LD based on staff time per session and number of cycles completed. Information on VIG costs was estimated using data reported by VIG practitioners between the 3-month and 6-month follow-up. Practitioners reported spending an average of 2.4 h per week (ranging from 1 to 4) on VIG, and 14 weeks from start to finish (range 5 to 29). NHS AfC bands ranged between 2 and 8. The mean cost per session was £153.35 (SD = £80.94) while the mean cost per cycle was £306.70 (SD = £161.87). The total cost of all VIG cycles per participant ranged from £378 to £1696 (mean = £852.00, SD = £430.55) based on the number of sessions they had completed. Meanwhile, for the 14 practitioners reporting, the average staff cost per hour was £63.71 (SD = £13.21).

To contextualise the cost of VIG-LD, we estimated TAU intervention costs using published literature [see Table S5 in Supplementary material 1]. According to our survey of specialist mental health services (*n* = 66), the two most frequently offered interventions were Applied Behaviour Analysis (£141.09 per session) and Cognitive Behavioural Therapy, which costs approximately £59.67 per face-to-face session and £64.83 per hybrid session.

#### Additional outcomes

Table 5 presents descriptive statistics on parent-reported measures at each of the three surveys. Overall, the means and standard deviations show little change over time, with the exception of closeness scores which increased for the VIG arm at 3 months and remained at that level at 6 months. The TAU survey where 66 specialist mental health services (similar to the study sites in the RCT) took part indicated that just 15% of these services (*N* = 10) had offered any type of video feedback intervention in the past 12 months, and this was mostly to families on the active caseload (with only 1.5% of services offering video-feedback interventions to families on referral or waiting lists). We considered not just VIG, but also interventions with a similar theory of change and/or use video as a therapeutic tool (VIG, VIPP, VIPP-SD, Marte Meo, VPCI, PACT). Findings suggested that interventions like VIG are not part of the typical provision in such services. TAU data collected in the RCT indicated that no video-feedback interventions (VIG or others) were made available to participants in the TAU arm. Data collected in the *n *= 66 survey indicated that the 3 most frequently offered supports are: psychoeducation, Applied Behaviour Analysis and Cognitive Behaviour Therapy. Data from this trial (3-month follow-up) indicated that the most common TAU was psychoeducation.

**Table 5:** Descriptive statistics on parent-reported measures per arm

	Group (*n*)	Baseline	3 months	6 months
CPRS conflict^1^	VIG + TAU (11)	23.46 (6.67)	22.73 (6.71)	23.73 (6.48)
	TAU (10)	25.30 (6.52)	22.80 (6.97)	24.80 (8.15)
CPRS Closeness	VIG + TAU (12)	24.50 (7.09)	27.08 (3.55)	27.08 (4.81)
	TAU (11)	25.73 (5.04)	24.64 (5.14)	25.27 (4.43)
APQ Positive parenting	VIG + TAU (12)	20.42 (2.11)	20.17 (2.04)	20.92 (3.42)
	TAU (10)	20.18 (1.89)	18.91 (1.38)	20.36 (2.54)
APQ Inconsistent discipline	VIG + TAU (12)	18.83 (1.19)	19.42 (2.31)	19.58 (3.12)
	TAU (10)	18.90 (2.42)	18.70 (2.00)	18.10 (2.23)
PHQ-4 Psychological distress	VIG + TAU (11)	3.73 (3.00)	4.90 (2.51)	4.64 (3.11)
	TAU (11)	4.36 (3.17)	4.64 (3.20)	2.64 (2.91)
BAP efficacy	VIG + TAU (12)	26.33 (7.18)	29.42 (4.54)	29.17 (6.10)
	TAU (11)	22.55 (9.02)	26.09 (6.44)	25.00 (6.75)
Vineland-3 Communication^2^	VIG + TAU (15)	50.33 (23.23)	n/a	46.67 (22.13)
	TAU (15)	37.13 (20.25)	n/a	34.13 (19.96)
Vineland-3 Socialisation	VIG + TAU (15)	51.27 (17.44)	n/a	52.20 (18.48)
	TAU (15)	45.00 (14.88)	n/a	48.27 (14.91)
DBC2 Total Raw	VIG + TAU (15)	93.60 (25.91)	n/a	91.73 (24.32)
	TAU (15)	87.60 (26.78)	n/a	82.40 (33.81)
DBC2Total T-score	VIG + TAU (15)	73.42 (11.01)	n/a	72.62 (10.33)
	TAU (15)	70.87 (11.38)	n/a	68.66 (14.36)
DBC2 Disruptive T-score	VIG + TAU (15)	64.02 (11.29)	n/a	63.33 (8.86)
	TAU (15)	63.12 (9.50)	n/a	62.01 (13.53)
DBC2 Self-Absorbed T-score	VIG + TAU (15)	74.97 (11.50)	n/a	74.45 (10.68)
	TAU (15)	76.15 (12.76)	n/a	72.74 (13.04)
DBC2 Communication Disturbance T-score	VIG + TAU (15)	66.70 (10.46)	n/a	67.20 (12.44)
	TAU (15)	61.33 (12.47)	n/a	60.66 (13.80)
DBC2 Anxiety T-score	VIG + TAU (15)	69.10 (15.81)	n/a	70.24 (15.17)
	TAU (15)	63.78 (13.09)	n/a	61.88 (14.38)
DBC2 Social Relating T-score	VIG + TAU (15)	63.77 (7.25)	n/a	61.48 (6.41)
		61.06 (10.97)	n/a	58.77 (11.17)

^1^*CPRS* Child-Parent Relationship Questionnaire [44], *APQ* Alabama Parenting Questionnaire [45], *PHQ-4* Patient Health Questionnaire[46], *BAP* Being a Parent scale [47], *Vineland-3* Vineland Adaptive Behaviour Scale Third edition [23], *DBC* Developmental Behaviour Checklist 2[26]

^2^Vineland-3 was assessed at screening and 6-month follow up

No safety incidents were reported during the trial.

Finally, Supplementary material 1: Tables S6 and S7 include findings from the process evaluation with regard to the acceptability of the study design by participants and clinicians, respectively [see Supplementary material 1]. A notable finding was parents’ suggestion that research in this area was evidence that ‘somebody cares’ for these families (“I feel like somebody cared for us…I feel like I am not alone”; P8). Parents shared that the study provided a sense of personal support, empowerment, and hope for the future, as well as increased knowledge and connection.

## Discussion

The present study is the first randomised evaluation of VIG with parents of children with intellectual disability, and one of just three feasibility evaluations of VIG in the UK, both other ones with parents of infants [19, 20]. The present study was designed to determine whether it is possible to conduct a definitive trial of remotely delivered VIG with this group of families. Two of the three primary feasibility objectives were met within the amber zone (recruitment rate and intervention adherence) and one within the green zone (study retention), supporting the feasibility of proceeding with a definitive trial of remotely delivered VIG for families of children with intellectual disability referred to specialist mental health services.

### Limitations

In England, there is large variation in the types of interventions that could be made available to children with intellectual disability and their families within specialist mental health services [5]. While qualitative findings supported the acceptability of VIG, there are no data on how many parents referred to such services may benefit from video-feedback interventions. The study managed to recruit sites with clinical experience of delivering VIG and sites without (2 versus 3, respectively), which may have introduced selection bias to the design. As we demonstrated that the majority of such teams do not offer VIG or video-feedback interventions more broadly, it remains uncertain whether clinical sites without existing VIG experience would be interested in adopting VIG or signing up to a trial; the current feasibility did not provide evidence on this, recruiting a small number of sites through convenience sampling. The profile of participants recruited is consistent with what is known for these families from population-representative or large-scale cohort studies [38–40], albeit levels of poverty were very high, despite levels of employment within the family, which may be related to the recent cost of living crisis. The parents recruited had children with severe intellectual disability (as measured by Vineland-3), and clinically significant levels of emotional and behavioural problems (as measured by DBC-2), consistent with the profile of children referred to such services. The study managed to recruit a higher percentage of parents from an ethnic minority background (27.5%) compared to the 2021 census population profile and to community-recruiting cohort studies [38] which may have resulted from increased recruitment efforts to capture under-represented groups.

### Implications for progression

The study’s independent Steering Committee and the Parent Carer advisory group provided strong support for progression to a final trial. We had had pre-determined that major changes would be needed to the recruitment approach if the recruitment target was within the amber zone but below the critical value of 42. Having consented 44 parents and randomised 40, substantial amendments are proposed drawing on evidence from the process evaluation (Supplementary material 1: Tables S6 and S7) and consultations with the Steering Committee and PCAG: to widen the recruitment pool, we will need to extend the upper age range of the children (which is also consistent with referrals typically accepted by the clinical sites), expand sites to include other settings where mental health support is provided (special schools or paediatric services with more informal mental health/behavioural pathways—consistent with NHS England’s published priorities for 2025/26 that suggest greater reliance on community systems to reduce pressures on specialist hospital services), and increase the resource available within each site to recruit participants. The fact that recruited participants came from the full spectrum of the referral pathway (referral, waiting list and active case load) is promising. Clinicians’ recognition that VIG can be offered not just to those on the active case load (in the process evaluation) is important for widening the pool of eligible participants. However, as this approach is incongruent with services’ clinical pathways—where interventions are offered only after in-depth assessment, we will need to work with local PIs to ensure recruitment takes place across all referral stages.

Adjustment to the intervention length period (allowing for longer than 12 weeks) was proposed as a way to increase VIG completion in these families where frequent instances of child ill health and other demands on families’ schedules mean that VIG sessions cannot always be scheduled weekly. As a longer term (12-month) follow-up will have to be added to evaluate maintenance, the 3-month follow-up could be eliminated to reduce burden (and retain three measurement time points). The high attrition seen for the telephone interviews suggests these are an additional hurdle to data collection. HE measures should be incorporated to the online surveys which had excellent completion levels. A more complete HE evaluation in the final trial should include a health-related quality of life measure for the parent; this was not included here as there is already evidence that it is feasible to capture such data from parents of children with intellectual disability [28, 41].

Findings across qualitative data raised questions about the suitability of the DBC-2 as a likely primary outcome in a future RCT as parents suggested that long, repetitive measures (DBC-2 and Vineland-3) are a burden. Despite the high completion rate of the DBC-2, parents and clinicians’ perception of VIG effectiveness focused on increased parent self-efficacy and improved parent–child relationship quality [see Supplementary material 1: Tables S1, S2 and S7 in Supplementary material 1], which are immediate outcomes of VIG, while child behaviour problems or mental health symptoms are more distal outcomes in the causal pathway. Consistent with this interpretation, the pattern of descriptive effect size estimates differed across outcomes with smaller estimates for the DBC (Hedges *g* = 0.12, 95%CIs − 0.27, 0.51) compared to parent–child closeness (Hedges *g* = 0.91, 95%Cis = 0.22, 1.60). The difference in magnitude of these estimates is consistent with the VIG logic model where changes in the parent–child relationship quality are the primary target with any changes in children’s behaviours that challenge being the more distal outcome. These estimates are reported to contextualise outcome selection rather than to indicate intervention efficacy, in line with the feasibility design. Parents expressed a preference for reporting positively oriented outcomes, which is consistent with the nature of VIG as well (strengths-based). In response to these findings, there was very strong support from the PCAG to consider parent–child relationship quality or parenting efficacy as the primary outcome, while still measuring child emotional and behavioural problems. For the latter, a measure other than the DBC-2 might be more suitable. Taken together, the study findings generated sufficient insight into the preference of the study participants with regard the primary outcome for a future RCT [42]. Parent–child relationship quality has been shown to be a risk/protective factor for child behaviours that challenge. A recent meta-analysis indicated that interventions offered to parents of children with intellectual disability to improve their relationship with their child lead to large improvements in parent–child relationship quality [12].

## Conclusion

This study successfully met its progression criteria, suggesting progression to a full RCT would be warranted. By addressing identified barriers and leveraging the intervention’s strengths, future research can further validate the potential of remote VIG to improve outcomes for families of children with intellectual disability. These findings would contribute to the development of scalable, evidence-based interventions that support families while reducing strain on specialist services.

## Supplementary Information

Additional file 1. Supplementary Table S1. Illustrative quotations of acceptability of VIG by parent participants mapped to each of the seven theoretical constructs of acceptability as defined by Sekhon et al. Supplementary Table S2. Illustrative quotations of acceptability of VIG by practitioners mapped to each of the seven theoretical constructs of acceptability as defined by Sekhon et aSupplementary Table S3. Parent-reported access barriers and facilitators, separated into overarching sub-themes. Supplementary Table S4. Clinician-reported implementation barriers and facilitators, separated into overarching sub-themes. Supplementary Table S5. The cost per session of other treatments that make up TAU based on published literature. Supplementary Table S6. A thematic analysis of the parent-reported evaluations of VIG-LD trial design, methods, and delivery. Supplementary Table S7. A thematic analysis of the clinician-reported evaluations of VIG-LD trial design, methods, and delivery.

Additional file 2. CONSORT checklist.

## Data Availability

The datasets used and/or analysed during the current study are available from the first author on reasonable request.

## Abbreviations

AFC: Agenda for Change
AVIG: Association for Video Interaction Guidance in the UK
CAMHS: Child and Adolescent Mental Health services
DBC: Developmental Behaviour Checklist
EOI: Expression of Interest
GBO: Goal Based Outcome
IQ: Intelligence quotient
ISRCTN: International Standard Randomised Controlled Trial Number
LD: Learning disability
MRC: Medical Research Council
NHS: National Health Service
PACT: Paediatric Autism Communication Therapy
PCAG: Parent Carer Advisory Group
RCT: Randomised controlled trial
TAU: Treatment as usual
TFA: Theoretical Framework of Acceptability
UK: United Kingdom
VIG: Video Interaction Guidance
VIPP: Video feedback Intervention to promote Positive Parenting
VPCI: Video Parent–Child Interaction
