# Supplementary material for: A feasibility randomised trial of remotely delivered Video Interaction Guidance for parents of children with intellectual disability referred to specialist mental health services
Source: Pilot Feasibility Stud. 2026 Mar 11;12:50. doi: 10.1186/s40814-026-01800-2 (PMC13088628; doi:10.1186/s40814-026-01800-2)
Supplement: Supplementary file 1 — Additional file 1. Supplementary Table S1. Illustrative quotations of acceptability of VIG by parent participants mapped to each of the seven theoretical constructs of acceptability as defined by Sekhon et al. Supplementary Table S2. Illustrative quotations of acceptability of VIG by practitioners mapped to each of the seven theoretical constructs of acceptability as defined by Sekhon et aSupplementary Table S3. Parent-reported access barriers and facilitators, separated into overarching sub-themes. Supplementary Table S4. Clinician-reported implementation barriers and facilitators, separated into overarching sub-themes. Supplementary Table S5. The cost per session of other treatments that make up TAU based on published literature. Supplementary Table S6. A thematic analysis of the parent-reported evaluations of VIG-LD trial design, methods, and delivery. Supplementary Table S7. A thematic analysis of the clinician-reported evaluations of VIG-LD trial design, methods, and delivery. [file 40814_2026_1800_MOESM1_ESM.docx]

**Title**: A feasibility randomised trial of remotely-delivered Video Interaction Guidance for parents of children with intellectual disability referred to specialist mental health services

Supplementary Table 1. Acceptability of VIG by parent participants (Theoretical Framework of Acceptability[1])

| **Construct** | **Sub-themes** | **Examples** |
| --- | --- | --- |
| 1. **Affective Attitude**   (This construct refers to the emotional responses given by parents in the VIG-TAU arm, including both positive and negative affective states.) | **1.1 Emotional Engagement & Enjoyment** | *"a lot more positive" (P1)* |
|  |  | *"I love it... I really enjoyed it" (P5)* |
|  |  | *"I'm glad I did it and I think I got quite a lot out of it" (P7)* |
|  |  | *"I quite enjoyed it in the end" (P7)* |
|  | **1.2 Curiosity & Interest** | *"I'm quite interested to know what last night's one was like" (P4)* |
|  |  | *"it was quite interesting." (P7)* |
|  | **1.3 Shared fun & bonding** | *"I'd be like ok [son name] lets go upstairs for our meeting and then he would get excited, and he dropped what he had, he'd hold my hand and go upstairs, because he really loved it as well...So he was really excited and he was really interacting really well and we had a lot of fun...It's funny to think how something like VIG could be fun, but it actually was. He was just having a ball”. (P1)* |
|  | **1.4 Discomfort & self-consciousness** | *"I think having someone not, you know, not judgy, but I mean effectively, it's sort of judgy and yeah, that is awkward...I didn't feel judged.... I just felt he was observing…I found it uncomfortable, but I didn't find it like hugely uncomfortable” (P7)* |
|  |  | *“I felt quite self-conscious, I'm not a centre of attention kind of person” (P7)* |
| 1. **Burden**   (This construct refers to the effort involved in participating in VIG, capturing logistical challenges and ease of integration) | **2.1 Logistical Challenges and Physical Effort** | *"That was the awkward thing, getting everything in the camera shot …It took forever trying to find a position that worked." (P5)* |
|  |  | *"It was tricky sometimes... it's always going to be tricky with [child name]." (P7)* |
|  | **2.2 Integration into Routine with Ease** | *"I could find a suitable time and then it wasn't overly onerous." (P1)* |
|  |  | *"It's not really been too much of an issue for me... they're things I would be doing or wanting to do with him anyway." (P4)* |
|  |  | *"It’s fine. So I don’t work... that wasn't a problem at all." (P7)* |
| 1. **Ethicality**   (This construct reflects whether VIG aligned with parents' values) | **3.1 Ethical Fit with Child's Needs** | *"This would only work online because [child name] wouldn't be able to relax properly." (P1)* |
|  | **3.2 Discomfort with Self-Observation** | *"I don't want to watch myself... it’s awkward…I particularly didn’t enjoy... when you play back me and [child name] together." (P7)* |
| 1. **Intervention Coherence**   (This construct looks at how well parents understood the purpose and process of VIG.) | **4.1 Clear Understanding of Intervention Goals** | *"It's about what do you feel like you're getting out of this interaction, what is your child getting out of this interaction and allowing you to pinpoint what could be better. You know it's about you being able to seek some help in your interactions or noticing a particular behaviour within your child****"*** *(P4)* |
|  | **4.2 Initial Confusion About the Process** | *"I thought it was more like a therapist would review us and tell us what to do next time." (P1)* |
|  |  | *"I've no idea what I'm watching or what you think I'm doing." (P7)* |
| 1. **Opportunity Costs**   (This construct explores the extent the value, benefits or gains must be given up to engage with VIG) | **None Reported** | |
| 1. **Perceived Effectiveness**   (This construct captures how effective parents found VIG in improving their interactions with their children.) | **6.1 Increased Awareness of Child’s Abilities** | *"He’s a lot more verbal than I thought... it wasn't just babbling but actually words." (P1)* |
|  | **6.2 Boosted Parental Confidence** | *"I learned a lot about myself as a parent that I shouldn’t be hard on myself." (P5)* |
|  |  | *"It’s rewarding to know what you’re doing right." (P5)* |
|  |  | *"[VIG] does help you to be reflective without being worried about being judged which I think is really good." (P4)* |
|  |  | *"It alleviated some of that mum guilt." (P3)* |
|  | **6.3 Struggles with Self-Reflection** | *"I’ve got no idea what I'm watching or what you think I'm doing." (P7)* |
| 1. **Self-Efficacy**   (This construct examines how confident parents felt in applying the insights from VIG to their future interactions) | **7.1 Sustained Confidence and Application** | *"VIG helps you stop and think... it becomes a part of your weekly routine." (P4)* |
|  |  | *"I'm a little bit more confident... I feel like I can do it." (P5)* |
|  |  | *[I think to myself] “you’re doing better than you think. Calm down. So I think I'm a bit more chilled" (P5)* |
|  |  | *"I feel like it is definitely something that even after the cycles are finished, you are helped" (P4)* |
|  |  | *"He comes to me more because I let him have that moment...I'm going to carry it on" (P5)* |
|  | **7.2 Limited Confidence in Broader Application** | *"Interviewer: “Do you think that anything that you might have talked about or discussed or learned in VIG may be helpful in other areas of your life?” Parent: “I don't think so, no." (P7)* |

Supplementary Table 2. Acceptability of VIG by VIG practitioners (Theoretical Framework of Acceptability[1])

| **Construct** | **Sub-themes** | **Examples** |
| --- | --- | --- |
| 1. **Affective Attitude**   (This construct explores the emotional responses of staff to delivering VIG, reflecting both positive engagement and nuanced preferences. | **1.1 Enthusiastic and Positive Emotional Engagement** | *"Hugely positive... I really came out of the sessions just absolutely buzzing." (S1)* |
|  |  | *"It was brilliant." (S7)* |
|  |  | *"I absolutely love VIG." (S4)* |
|  | **1.2 The Beauty and Emotional Impact of VIG** | *"It’s just so beautiful." (S2)* |
|  |  | *VIG helped mum to 'find some joy again, and it was really beautiful to be part of that.'" (S6)* |
|  | **1.3 Powerful Emotional and Reflective Impact** | *"We’ll find the best moments of that attunement... it’s wonderful." (S2)* |
|  |  | *"It was really, really powerful." (S1)* |
|  | **1.4 Appreciation for the Intervention, but Preference for In-Person Delivery** | *"I love VIG. I absolutely love VIG... but I will really try to do it in person." (S4)* |
| 1. **Burden**   (This construct looks at the effort required to deliver VIG, from both logistical and cognitive perspectives.) | **2.1 High Effort and Time Commitment** | *"It was challenging... logistics of taking the video after school, childcare, and siblings." (S1)* |
|  |  | *"It was a nightmare... more time intensive than I'd originally anticipated." (S2)* |
|  | **2.2 Technical Challenges** | *"It looks like you’re filming the whole picture but... it might have chopped off their heads."* |
|  | **2.3 Flexible and Low-Effort Delivery** | *"It was very easy." (S9)* |
|  |  | *" I think in terms of the load on the clinician, it was absolutely fine...seeing how quickly you could get the benefits from those sessions with helpful." (S1)* |
|  |  | *"It’s cost-effective... you just need an Internet connection." (S9)* |
| 1. **Ethicality**   (This construct addresses whether VIG aligns with the staff’s professional and personal values.) | **3.1 Alignment with Values of Empowerment and Attachment** | *"How can you not love an intervention that puts the parent and child at the centre?" (S2)* |
|  |  | *"Attachment is something I’m very keen on." (S3)* |
|  | **3.2 Person-Centred Approach for Diverse Parent Needs** | *"We had to make it person-centred... some parents won’t be that reflective." (S3)* |
|  |  | *“[VIG is] going to look however it looks depending on the developmental level of the kid, so the flexibility is already there I think" (S6)* |
|  |  | “It really must fit in with the lives of families as they live it in order for them to be effective, and I think VIG does all of that.” (S9) |
|  | **3.3 Ethical Concerns with Remote Delivery** | *“He really liked to be outside and she didn't have enough Internet, it didn't reach the bottom of their garden...she had to keep him inside and he'd get a bit ratty inside" (*S6) |
| 1. **Intervention Coherence**   (This construct examines how well staff understood the purpose and process of VIG) | **4.1 Clear Understanding and Training Support** | *"The training gave a good background... a better understanding of what VIG is." (S7)* |
|  |  | *"The role-playing that comes within the training was really helpful." (S9)* |
|  | **4.2 Initial Confusion and Gaps in Understanding** | *"Initially the staff team really struggled to understand how this could be helpful… It was a culture shift in thinking." (S2)* |
| 1. **Opportunity Costs**   (This construct examines whether the staff perceived any cost due to delivering VIG) | **5.1 Additional time resource required to learn new intervention** | *“I shared with my team that I'm finding it hard, and I asked for time, one more remote day in my week so that I can spend more time on VIG…But after the first review, first cycle, I felt I can do this without spending much time and the time I'm spending has reduced, so I became confident like I have time and I communicated this with my team.”  (*S3) |
| 1. **Perceived Effectiveness**   (This construct explores how effective staff felt VIG was in achieving its intended outcomes.) | **6.1 Transformative for Families and Insightful for Practitioners** | *"It feels enormously transformative in their day-to-day lives." (S1)* |
|  |  | *"It gives everyone insight... the parent, the practitioner, and the therapist... but what’s the next piece" (S4)* |
|  |  | *“Without VIG I would never have been able to have convinced mum that she did have a lot of gifts and so did her daughter. It was just such a beautiful thing to do with them.”  (S8)* |
| 1. **Self-Efficacy**   (This construct explores how confident staff felt in delivering VIG in the future.) | **7.1 Increased Confidence and Skill Transfer** | *"I’ve definitely taken some of those things into my practice." (S1)* |
|  |  | *"I feel more empowered going into sessions." (S9)* |
|  | **7.2 Gaps in Team Support** | *"I do worry about VIG just not being like any kind of intervention we’re delivering to families." (S3)* |

Supplementary Table 3. Parent-reported barriers and facilitators to accessing VIG offered remotely

| **Theme** | **Primary sub-theme** | **Second sub-theme** | **Quote** |
| --- | --- | --- | --- |
| **Barriers to remotely delivery VIG** | 1. **Difficulties engaging in core components of VIG** | **-** | *"I particularly didn't enjoy when you play back me and [child name] together. Hideous....I don't want to watch myself, it’s awkward…I don't think anything is wrong with finding it difficult. I think I just found it difficult” [P7]* |
|  | 1. **Challenges to Child Engagement** | **-** | *"I didn’t practice anything because [child’s name] was high, high, high. And [clinician] said ...when he is going to start his medicine, he will be calm ...we gonna start everything then" [P3]* |
|  |  |  | *“[He] didn't want to because they wanted to see us do an activity together and he just wasn't interested... [he] very much does things on his own terms." [P11]* |
| **Facilitators of remotely delivery VIG** | 1. **Home-Based Intervention reduces additional stressors for child** | **-** | *"Online was much better I think, delivered this way for us...because he was in a comfortable environment." [P1]* |
|  | 1. **Easily Integrated into routine Daily Functioning** | ***2.1 Flexible & Convenient Scheduling*** | *"I didn't have to try and fit it into a certain time. At the end of the day or before dinner time, I could find a suitable time and then it wasn't overly onerous." [P1]* |
|  |  |  | *"I always worried about when we would fit it in, but it's been so flexible, so it's been really easy" [P4]* |
|  |  |  | *"See that worked for me because with us having so many appointments...it just helped because we could pick a time...And then on the other day talking about it when they’re at school, it's easy to find a bit of time so that's good" [P5]* |
|  |  | ***2.2 No Travel Requirement*** | *"I actually found for me it works better because one, I'm not going out to say the hospital or the doctor's for an appointment" [P5]* |
|  |  |  | "If you’ve got to go somewhere, it's a bit harder sometimes to try and find that time to get there" [P6] |
|  |  |  | *"I didn't have the travel time in between" [P1]* |
|  | 1. **Minimal Intrusion on Interaction** | **-** | *"Once the cameras rolling and she takes the video down, even though she's recording, so she's there but in the background, I think that helps because then you just think your phones up but not on sort of thing because it’s just a black screen." [P5]* |

Supplementary Table 4. Clinician-reported barriers and facilitators to offering VIG remotely

| **Theme** | **Primary Sub-theme** | **Second Sub-theme** | **Quote** |
| --- | --- | --- | --- |
| **Barriers to remotely delivery VIG** | 1. **Preference for In-person or Hybrid Delivery** | ***1.1 Perceived Parent Prefer In-Person*** | *“Some of them really love that human contact as well. And sometimes that might be the only adult that they get to speak to that day” [S2]* |
|  |  | ***1.2 Staff Preference for Hybrid*** | *“Shared reviews online, absolutely fine……[but] when I was supervising people, they were saying that would be the only thing they would change, the flexibility to go out to the house to film” [S2]* |
|  |  |  | *“I think it's limited how much you can do when you're not physically with them… I would have really liked to be a little bit more hands on and help them to get their best interaction.”  [s4]* |
|  | 1. **Parent and Child Capacity to Engage** | ***2.1 Presence of Complex Care Issues and External Stressors*** | *“I know our population; we don't have any service user with just one diagnosis. Most of them have minimum 3 diagnosis plus strained attachment, physical health [issues] and I was hesitant…So during that time when we do the video recording, [could] the child be settled and play with the parent?” [S3]* |
|  |  |  | *“It just got really difficult in the family home, social care issues, medication issues. She just asked to put things on pause" [S6]* |
|  |  |  | *"If there was a really complex case with really challenging behaviour and a family was almost in crisis, just firefighting all the time, I’m not sure whether I'd be comfortable then" [S7]* |
|  |  |  | *“I think that's impacted… one of the young people who just hasn't managed to get the three cycles in and I was just like, we've got a national shortage of ADHD medication” [S2]* |
|  |  |  | *“They're trying to get a young person who has got a learning disability, all of the participants actually had Autism and or ADHD, trying to get them just to do an activity for 10 minutes in one place, with good quality filming was a challenge” [S2]* |
|  |  | ***2.2 Parent Emotional State*** | *“People's typical experience of coming into an intervention is to be told what to do differently.” [S2]* |
|  |  |  | *"She asked for a pause because she just said that I don't have the headspace to try and do this” [S6]* |
|  | 1. **Technological Difficulties** | ***3.1 Compromised Recording Quality*** | *"It looks like you're filming the whole picture, but then when you go into your video, it might have chopped off their heads. So obviously that's quite critical" [S6]* |
|  |  |  | *“There is too much going on in the background and you know, just life basically doorbell going all sorts of things happening, people calling round.” [s8]* |
|  |  | ***3.2 Inconsistent Internet*** | *"Stressful in that Teams doesn't always work" [s6]* |
|  |  |  | *“Technical difficulties have been a bit of a challenge…like internet difficulties” [s8]* |
|  |  | ***3.3 Internet Limiting Activity Choice*** | *"He really liked to be outside and she didn't have enough Internet, it didn't reach the bottom of their garden...she had to keep him inside and he'd get a bit ratty inside" [s6]* |
| **Facilitators of remotely delivery VIG** | 1. **Parent Preference for Online Delivery** | **-** | *"It was a case where it would have been more than feasible to do it face to face...but she was like no, all virtual please, just so much more easier to manage." [S1]* |
|  |  |  | *"I think certainly parents seem to prefer video meetings if the child isn't involved, it just reduces the amount of disruption to their lives." [S2]* |
|  |  |  | *“Parents generally do want zoom. They do like that flexibility” [S5]* |
|  |  |  | *“Oh if it was face to face parents would have found it more difficult…For the families that I'm working with, I would say they have no issues with offering this remotely.” [S3]* |
|  | 1. **Convenient & Enables Naturalistic Interactions** | **-** | *"The comfort of their home and secondly, the flexibility to choose when they could have that interaction made it easier for parents to engage." [S9]* |
|  |  |  | *"Because you’re literally filming what's there and there's no prescribed activity that they need to be doing, so the flexibility is already there" [S6]* |
|  |  |  | *“What I could see is where they may have felt a bit overwhelmed by being sat with somebody in a clinical room, their computer gave enough of a filter for them to really engage” [S9]* |
|  |  |  | *"The phone being in the room is less obvious than a human filming to capture those kind of like natural interactions between children and the parents." [S7]* |
|  |  |  | *"In terms of if we were to go out into people's houses, there's a possibility of us being more of a distraction or a trigger, if children find it really difficult to tolerate new people" [S6]* |
|  | 1. **Perceived Effective Use of Resources** | **-** | *"Obviously delivering it virtually is enormously beneficial, both in terms of a clinician planning your time and in terms of the family staying in. Particularly for a service like ours, we can be three hours away from our families" [s1]* |
|  |  |  | *“It allows flexibility, reduces resource, time for travel, see more families, reduces demand on clinical space. It provides privacy, a confidential space which could not be offered in person” [S5]* |
|  |  |  | *“I think it's cheap, convenient, flexible. I think in everything that parents have to do throughout the day, including attend to the needs of their children, I think, fits quite well” [S9]* |
|  |  |  | *“You can see so many more patients. You can do it privately, confidentially, book a room generally to see everyone. So that helps.”   [s5]* |
|  | 1. **Accessible to Families** | **-** | *"You know, they travel enormous distance to come and see us, and it wouldn't be feasible to deliver interventions in person like that unless they were being delivered virtually" [S1]* |
|  | 1. **Enhances Clinical Understanding of Family** | **-** | *“I feel online is perhaps more helpful because it happens at home, in a setting that's natural to parent and child, so we see both the order and the disorder at home. “[S9]* |

Supplementary Table 5. Costing interventions offered by specialist mental health services.

| **Therapy Type** | **Cost per session** |
| --- | --- |
| Hanen Programme | £266.18[2] |
| Paediatric Autism Communication Therapy (PACT) | £264[3] |
| Video-feedback Intervention to promote Positive Parenting (VIPP or VIPP-SD) | £261.92[4] |
| Video Interaction Guidance (VIG) | £153.35 |
| Psychotherapy | £152[5] |
| Applied Behaviour Analysis | £141.09[6] |
| Incredible Years | £130.75[7] |
| Counselling | £126.67[5] |
| Family Therapy | £71.75[5] |
| Cognitive Behaviour Therapy (CBT) - Hybrid | £64.83[8] |
| Cognitive Behaviour Therapy (CBT) - Face to Face | £59.67[8] |
| Sleep Therapy | £52.6[9] |
| Play Therapy or Theraplay training | £50.7[5] |
| Psychoeducation | £43.76[10] |
| Stepping-Stones Triple P | £32.89[11] |
| Lego Therapy | £9.57[12] |

Supplementary Table 6. Thematic analysis of parent responses to evaluation of VIG-LD trial design, methods and delivery.

| **Stage of trial** | **Primary Theme** | **Secondary Sub-themes** | **Quotes** |
| --- | --- | --- | --- |
| **Overarching Trial Experience** | 1. **Support, Value and Hope through participation in the VIG-LD trial** | ***1.1 Supportive Interactions and Empathy from researchers*** | *"But I know you're a mum so it's like I know you can understand where we're coming from...I just think that empathy and warmth meant a lot definitely because you’re nervous and you don't know what to expect" (P5)* |
|  |  |  | *“The people who I've spoke to on the phone and even through emails have been amazing. And if I've needed any questions or anything, everyone's been really, really nice.” (P9)* |
|  |  | ***1.2 Feeling Valued, Helped and Hope*** | *"I feel like somebody cared for us…I feel like I am not alone” (P8)* |
|  |  |  | *"I've enjoyed being part of the study. It's made me feel important, like me and my family’s experience matters, which is nice. So thank you" (P1)* |
|  |  |  | *“I feel that things will move in the future [because of the trial]" (P8)* |
|  |  |  | *“I felt excitement at that we might get some help and a bit of relief as well, that there's actually something out there that can help” (P2)* |
|  |  |  | *"I just really appreciated being a part of a trial that I think has got major potential to really help a lot of different families" (P1)* |
|  |  | ***1.3 Empowerment and Knowledge Building*** | *"I've enjoyed doing the research because like I said, it's gave me a bit of more knowledge " (P6)* |
|  |  |  | *" We were told every step of the way that we can pull out at any time." (P11)* |
|  |  | ***1.4 Personal Enjoyment*** | *"I've really enjoyed it" (P5)* |
| **Screening & Recruitment** | 1. **Mixed experiences of Clarity, Support & Uncertainty in Recruitment & Screening** | ***2.1 Positive Experiences with Supportive Communication & Personal Connection*** | *"When I talk to someone, I feel better, you know, yeah...They told me what was going to happen." (P3)* |
|  |  |  | *"When you rang me up, that phone call, I’d say put my mind at ease…you told me what it was all about…you got to know me " (P5)* |
|  |  |  | *"I think that helped as well. It was good to be able to talk about him and be listened to. It was nice" (P6)* |
|  |  |  | *"It made me feel comfortable and it was nice to have someone listen to me because it feels like, in Services, there's no one that listens to you, really. So that was nice. " (P2)* |
|  |  |  | *“Sometimes it's nice to talk and nice to go through things when you are struggling” (P9)* |
|  |  |  | *"It meant that you were not just being left. You had people contacting you. I think that was a good thing. To be honest, it was helpful." (P6)* |
|  |  | ***2.2 Clarity and Utility of Visual and Written Materials*** | *"I did watch the video which was good to watch. It all made sense"* |
|  |  |  | *"I remember watching the video...she was really positive about it, so that was good to hear from a parent." (P1)* |
|  |  |  | *"Everything was clear, and I was expecting all the things to happen this way, so everything was fine for me, and everything was organised and in order" (P8)* |
|  |  |  | *"To me it was fine...it made sense" (P2)* |
|  |  |  | *“It was fine." (P11)* |
|  |  |  | *“Yeah, yeah, pretty fine.” (P9)* |
|  |  | ***2.3 Expectations & Misconceptions about the Intervention*** | *Thought was like therapy. "I didn't gain a lot from the four-page thing really...I thought it was more like a therapy-based thing. Like I would get therapy through it, but when I got into the trial, I realised that it was more kind of parent led." (P1)* |
|  |  |  | *" I don't know if the [recruitment] information video was that helpful. It was either that or I just thought that it was gonna be a lot of constructive criticism...I didn't really know what rewatching some of the videos would be like, but it's been quite nice" (P4)* |
|  |  | ***2.4 Areas of Uncertainty and Confusion*** | *"I don't know how you got my details... I don't know how I was recommended" (P5)* |
|  |  |  | *"It wasn't clear for me what was going on...it was a lot of confusion. I didn’t know if it was because I was very exhausted at that time" (P10)* |
|  |  |  | *"I didn't really know what I signed up for you know, I didn't know what I let myself in for" (P7)* |
|  |  |  | *"I didn't sort of understand the whole research...I think I got a bit confused about it, but I obviously I understood it after a while, to fully grasp it." (P6)* |
| **Randomisation** | 1. **Mixed Expectation and Understanding of the Randomisation Process** | **3.1 Preparedness and Understanding of Randomisation** | *"It was very clear...I was very excited that I did get the VIG, that I fell into that group. That's really good, but then I also prepared myself that I was going to do the questionnaires anyway and it wouldn't have been of that like bigger deal" (P1)* |
|  |  |  | *“I think when you don't know what side of the study you're gonna be in, you try not to think about it too much. You don’t wanna get too hopeful, you know, and then be disappointed." (P4)* |
|  |  |  | *"Yeah, I got that, that was clear to me" (P7)* |
|  |  |  | *"From the beginning, everything was clear for me " (P8)* |
|  |  |  | *“Yeah, that's fine” (P9)* |
|  |  |  | *"Yeah and it was ok. It was ok for me." (P3)* |
|  |  | ***3.2 Confusion about randomisation*** | *"I thought if I've made an effort that I'd get picked" (P2)* |
|  |  |  | *"I wasn’t sure. I think just to give them more information. Maybe as to why someone would be in one group." (P6)* |
|  |  |  | *"no one told me that I was still in the study because the doctor said that I had not been selected for a group and he did not explain more." (P10)* |
| **Data Collection (Baseline, 3m, 6m & CASUS)** | 1. **Ease, Engagement & Frustration with Data Collection** | ***4.1 Perceived Ease & Accessibility of Surveys*** | *"I think it was fine because when you get the email and it says this is your survey, it always gives you an approximate time of how long it can take" (P4)* |
|  |  |  | *"I found it OK, I'll be honest. I didn't find it too bad."  (P6)* |
|  |  |  | *"They were dead easy to do. Yeah, I didn't have any problems with it...you know it’s quite fun talking about [child name] sometimes, you know, I quite like it" (P7)* |
|  |  |  | *“I can’t remember there being any problems it's just easy, straightforward” (P9)* |
|  |  |  | *"It all seemed quite straightforward." (P11)* |
|  |  |  | *"I've seen all these types of questions before…it was fine for me" (P1)* |
|  |  |  | *"It was easy for the surveys. Easy to get on to do them. I've never needed to put my ID number in... I don't recall ever struggling or thinking that any of the questions were not appropriate or anything" (P2)* |
|  |  |  | *" I think they're quite easy to fill out" (P4)* |
|  |  | ***4.2 Positive Impact of Incentives*** | *"The vouchers were good incentive. I mean, I would have done it regardless, but it comes in handy as well, doesn't it" (P2)* |
|  |  |  | *"The vouchers were brilliant incentives as well...it's nice to get something like that". (P1)* |
|  |  | ***4.3 Personal Reflection & Insight Through Surveys*** | *"I think that's sort of like what I was partially doing when I was doing the questionnaires, I was thinking a bit more about his needs" (P6)* |
|  |  |  | *"Through the questionnaires, I realised my son has made some good steps because I don't see my son in many of the questions, so this is a good thing. So, I learn more even about the situation of my son, even I get more knowledge" (P8)* |
|  |  | ***4.4 Language & Comprehension Challenges*** | *"There was no difficulty beside that sometimes my English is not very good, but I can understand, most of them I responded, and I found it quite interesting" (P8)* |
|  |  |  | *"Because English is not my first language, I had to take my time to answer and to know what I'm writing and what I'm choosing…[it] is difficult." (P10)* |
|  |  |  | *"So the questions were sometimes hard to understand because my English...The questions, sometimes I read and was confused, do you see? That’s why I prefer to talk to someone." (P3)* |
|  |  | ***4.5 Frustration with Question Relevance and Survey Length*** | *"Sometimes I had to really read what the question was asking because they were quite detailed and then some of it didn't apply particularly." (P1)* |
|  |  |  | *"The first one I felt like because we just had that conversation that a lot of it was covered and it was a bit too long… certain questions didn’t apply to (child 1 name) at all...there was a lot that didn't apply" (P5)* |
|  |  |  | *"My only thought with some of the questions was they just didn't apply...there wasn't really an option for just like, completely not even relevant " (P6)* |
|  |  |  | *"Yeah, it was quite very long…the questions repeated in different ways several time and I found it repetitive" (P10)* |
|  |  |  | *"There was a lot of questions" (P3)* |
| **Suggestions provided by parents to aid future trial design** | 1. **Parent Suggestions to Improve Study Design or Processes** | **5.1 Enhanced Communications & Check-Ins** | *"Make it clearer that it is random. So, no matter how much you engage, it doesn't mean you will get selected" (P2)* |
|  |  |  | *"I think maybe a call would be useful and just to sort of check in…because you could miss it in your emails really easily" (P2)* |
|  |  |  | *"I think just to give them more information. Maybe as to why someone would be in one group." (P6)* |
|  |  | **5.2 Expand Recruitment Channels** | *"Engage directly with special schools. Because I only heard about this through a paediatrician and otherwise, I would never have a clue what VIG was, or that you were doing this trial" (P1)* |
|  |  | **5.3 Tailor Questionnaire Content to reduce length and repetition** | *"Even just an option to say, you know, just not even possible, right" (P7)* |
|  |  |  | *"Say for instance you do the general questions you want answering, but then if you've got a verbal child, ask for the next sets are questions if your child is verbal? If yes, complete this section. If not, go straight onto this section and the same with the like toilet trained or a certain age" (P5)* |
|  |  |  | *"I think you should make the questionnaire less lengthy because most of parents, I believe, like me, have too much to do and don’t have very much time to give. If we keep repeating the same thing repeatedly—it makes you think like “oh, I don't want to go through this.” (P10)* |

Supplementary Table 7. Thematic analysis of clinician responses to evaluation of VIG-LD trial design, methods and delivery.

| **Stage of trial** | **Primary Theme** | ***Secondary Sub-themes*** | *Responses/ Quotes* |
| --- | --- | --- | --- |
| **Overarching Trial experience** | 1. **Professional Growth, Fulfilment, and Positive Impact on Families** | ***1.1 Enhanced Professional Development and Skill-Building*** | *A lot of them reached midpoint in the trial, which was fabulous. It supported their VIG training journey because of the trial. They've had more clients, they've had more cycles” (S2)* |
|  |  | ***1.2 Satisfaction and Enjoyment in Participating in the Intervention*** | *"Just really glad I've got to be a part of it. It's a nice intervention" (S7)* |
|  |  | ***1.3 Perceived Benefits for Families Involved*** | *“It was a benefit to the families as well because they're getting a VIG practitioner at a different stage in their journey, who's achieved that midpoint.”* (S2) |
| **Recruitment** | 1. **Perceived Challenges, Success, and Sensitivities in Screening & Recruitment** | ***2.1 Low Effort required from Clinical Team*** | *"The fact that other than sign posting families and then potentially maybe sending a follow up email and attaching something, then the study team very much took the lead and were available to answer questions and maybe encourage and do all of those things was phenomenal in terms of taking the load off clinicians" (S1)* |
|  |  | ***2.2 Dedicated Resource for Screening aids family engagement*** | *[Had it] “Originally we weren't getting many numbers through and I couldn't work that out actually because I expected a lot more people to come through from our families…then we got the assistant [psychologist] to contact everybody just to see if there's a bit more interest and that seemed to make a difference to how many people are expressing an interest to be part of the trial.” (S8)* |
|  |  |  | *[Had it]* ***“****I thought about eligible families within the service and had those initial conversations explaining the intervention [and] invited their interest…It worked.” (S9)* |
|  |  |  | *[Had it] “Yeah, that seemed to be a game changer.” [S8)* |
|  |  |  | *[Had it] "We had a really good response to it actually from what I remember. Like way more than we thought we were gonna get came flooding in…we had an assistant psychologist calling families " (S7)* |
|  |  |  | *[Needed it] “screening for families was time consuming, this is a lot. Should I be having to do this?” (S5)* |
|  |  |  | *[Needed it] “I wasn't attending those team meetings. That's a reflection I've got. That maybe I should have been going to more of those meetings in person rather than just trying to catch them in the office, to get it at the forefront of their minds, that this could be a really good addition to what they were already receiving”* *(S2)* |
|  |  |  | *[Needed it] "We needed an assistant psychologist. I think that's what we've learned...I can see the value in having someone working through the lists in a more routine way." (S2) [Context – Manager was only staff member involved in study recruitment.]* |
|  |  | ***2.3 Constraints within Clinical Service hindered Recruitment*** | *[Limited access to caseload] “What is challenging for a service such as ours is the fact most of the clinicians don't hold a case load...we're not able to just kind of go into a case load and pull families out…There's only a couple of others that have access to the waiting list in terms of being able to pull out families." (S1)* |
|  |  |  | *[overestimation of eligible families] “I don't know what percentage of the children and young people we are seeing have an intellectual disability, but I think maybe the volume was not what would have been useful to the study " (S1)* |
|  |  |  | *[overestimation of eligible families] Age range of eligibility was “difficult because most of our children are older teens with an Autism diagnosis and suspected LD”. (S5)* |
|  |  |  | *[Limited ability promote study to team / families] “our clinicians go out to the home environment…so actually putting posters up in a waiting room is redundant. We don't have social media channels… It's a little bit challenging for us to promote things”.  (S2)* |
|  |  |  | *[Team’s perceived ambivalence to new intervention] “They really took a little while to grasp it. Towards the end, when recruitment was obviously due to close, I think that's when the penny dropped for the team, and they were like oh now we can see it.” (S2)* |
|  |  | ***2.4 Sensitivity to Family Needs and Responses*** | *[Perceived gratitude for invite] "Because of the nature of our service, families often have a bit of a kind of sigh of relief, of being listened and validated too. So I think that that probably helps" (S1)* |
|  |  |  | *[Perceived indignation for invite] “about two or three parents, they were annoyed with our service, and I just think they thought it was insulting. Like, why are you ringing me up offering this when I'm waiting and things are really, really tough… And if you feel like you've been on a waiting list for a while, you're not maybe gonna be receptive in that way”* *(S2)* |
| **Delivery of VIG as prescribed through trial** | 1. **Balancing Structure & Flexibility in Delivering VIG** | - 1. ***Sufficiency of Intervention Structure & Cycle Duration*** | *"Three to five cycles, that doesn't seem like much and actually it was, it was absolutely enough, really clearly to me and the family, it was enough" (S1)* |
|  |  | - 1. ***Positive Impact of Supportive Leadership on Caseload Management*** | *" I feel like I was really well supported and not just from my supervisor, but from my clinical lead as well. She was really supportive about the study and saw the value in it ...she was really good at not allocating me 100 cases or not allocating lots of work alongside so that I had that breathing space" (S7)* |
|  |  | - 1. ***Scheduling Pressure and Caseload Juggling*** | *"So, when the VIG cycles were rolling at pace, if you like, they do sort of have to take over chunks of your diary and so yeah, you’re sort of having to prioritise those because you've got that kind of pressure of every week" (S6)* |
|  |  |  | *"It's always the issue with juggling caseloads and things like that. Just making sure that you've got that time in the time" (S7)* |
|  |  |  | *"The speed of trying to put the first appointment being within a week was hard" (S1)* |
|  |  | - 1. ***Unpredictability of Family Availability & Extended Timelines*** | *“There was so much frustration around, oh gosh, this intervention should have been completed by now. But families had cancelled, kids would get ill, holiday periods would come up.” (S2)* |
|  |  |  | *“You couldn't predict when participants would get picked up… It's very true to life with video interaction guidance in our service, things get in the way and then it drags out the intervention” (S2)* |
|  |  | - 1. ***Challenges with Protocol Flexibility & Preference for In-Home Filming*** | *"All three of them said, why can't I go to the house to film this? …I think if the protocol could be tweaked a little bit, shared reviews online, absolutely fine, that's not a problem." (S2)* |
| **Outcome Measure** | 1. **Enhanced Awareness, Confidence & Connection in the Parent-Child Relationship** | ***4.1 Increased Awareness & Understanding of Child’s Behaviour*** | *"[parent said] he is communicating with me all the time for different reasons and he's doing it in different ways...more than I thought he was...he didn't change necessarily what he was doing but it was just her recognising it." (S1)* |
|  |  |  | *“She [mum] realised* *he is giving me eye contact, he is communicating in his own way. And her goal was for him to communicate with her more… Especially the first session, the first review was very powerful with her.” (S3)* |
|  |  |  | *I really found that VIG was quite powerful when it captures the embodied, the nonverbal, the more somatic ways of communicating…it was these kind of things that they pointed out in the videos, they were pointing out non-verbal language, how they were sitting, the sounds they were uttering, and recognising the embodied communication between parent and child.” (S9)* |
|  |  | ***4.2 Validation of Parental Competence & Increased Confidence*** | *"The big thing for her was just recognising that what people were saying about her parenting was true because she couldn't believe that she was a good enough parent because her child was still having these outbursts. And at the end of it, she was like I am a good enough parent. These outbursts aren't because of me. Which I think was a really powerful message for her to hear.” (S6)* |
|  |  |  | *“Even though the system’s really stressed and the system's really anxious and all of these behaviours are getting worse, it's not to change the behaviour, but it's just actually to help the parents see that they've got that connection with their child. And that's the most powerful thing” (S2)* |
|  |  |  | *"The main thing for me, is just seeing that increasing confidence in her. It felt really transformative...it was much more around her confidence as a parent" (S1)* |
|  |  |  | *“She didn't directly reflect with words, but from her body language and everything, she is finding it hard to take the positives, to take the compliments…[but] in that sense I could see some difference in her, in the way she slowly started accepting the compliments, accepting her own skills.” (S3)* |
|  |  | ***4.3 Improvement in Parent-Child Relationship & Connection*** | *"She was just so low in confidence and just had a belief that her son didn't like her...then by the last shared review, she was like, oh, he really likes being with me. And he started coming to me more. And he'll come and grab my hand now, which he never used to do...she felt it had really built on their relationship. And yeah, it was massive." (S7)* |
|  |  |  | *"She was like, I feel more connected to him." (S1)* |
|  |  |  | *"[Mum said] Now I see and now I plan for us to have these one on one times together and we really enjoy them...I guess it's that positive reinforcement cycle. I feel more connected” (S1)* |
|  |  |  | *"She's feeling more confident and wants to be around him" (S7)* |
|  |  | ***4.4 Utility of Clinical Assessment Tools*** | *"Using the goals and looking at the scaling was good…It was like “I'm a three. I'd be absolutely over the moon to get to a 6” and then she's like, oh then eight, Oh my God." (S1)* |
|  |  |  | *“The ones we typically use in our service, is the parental stress scale questionnaire… but sometimes the stress can come from external factors, it's not necessarily the relationship with the child that's causing the stress… I think maybe you just need to devise a relational measure for VIG” (S2)* |
| **Suggestions when designing future VIG RCT** | 1. ***Staff Suggestions to Improve Future VIG Trial (5)*** | ***5.1 Increase Study Visibility Through Visual Reminders*** | *"I think something to go up in the clinic rooms would probably be helpful..., I think it would predominantly be there for staff. But absolutely, parents could see it as well." (S1)* |
|  |  | ***5.2 Clearer Guidance in Protocol on Contact Attempts by Clinical Staff*** | *“Clinicians weren't familiar with this, I think they forgot to revisit it with these parents because I think at that point they might have come on board …would the protocol allow for us to keep going back potentially to those already contacted” (S2)* |
|  |  | ***5.3 Flexibility in Delivery Methods & Timeframe*** | *"I'd like the flexibility personally, depending on a person’s situation because a lot of the anxiety around VIG was the technology, is it gonna work? I think you would take a lot of that anxiety away and you would just kind of go and catch up with somebody...But the fact that you've got that virtual option in those instances where, for example, you have a child where you may completely blow any chance of a positive interaction just by being there...being virtual could be even easier because you could go home and catch them later in an evening and it's like 20 minutes out of your evening versus at home visit" (S6)* |
|  |  |  | *"It’s hard to put a timeframe on it...we do get a lot of cancelled appointments once kids are sick and things like that" (S7)* |
|  |  |  | *“I think if it were delivered as an intervention within services, I think just the fact that it's being offered by a service with which the family is familiar would be enough to maybe make them feel comfortable to accept the intervention) (S9)* |

**Reference list**

1. Sekhon M, Cartwright M, Francis JJ. Acceptability of healthcare interventions: an overview of reviews and development of a theoretical framework. BMC Health Serv Res. 2017;17:88.

2. Roddy A, O’Neill C. The economic costs and its predictors for childhood autism spectrum disorders in Ireland: How is the burden distributed? Autism. 2019;23:1106–18.

3. Byford S, Cary M, Barrett B, Aldred CR, Charman T, Howlin P, et al. Cost-effectiveness analysis of a communication-focused therapy for pre-school children with autism: results from a randomised controlled trial. BMC Psychiatry. 2015;15:316.

4. O’Farrelly C, Barker B, Watt H, Babalis D, Bakermans-Kranenburg M, Byford S, et al. A video-feedback parenting intervention to prevent enduring behaviour problems in at-risk children aged 12-36 months: the Healthy Start, Happy Start RCT. Health Technology Assessment. 2021;25:1–84.

5. Mavranezouli I, Megnin-Viggars O, Trickey D, Meiser-Stedman R, Daly C, Dias S, et al. Cost-effectiveness of psychological interventions for children and young people with post-traumatic stress disorder. Journal of Child Psychology and Psychiatry. 2020;61:699–710.

6. Rodgers M, Marshall D, Simmonds M, Couteur AL, Biswas M, Wright K, et al. Interventions based on early intensive applied behaviour analysis for autistic children: a systematic review and cost-effectiveness analysis. Health Technology Assessment. 2020;24:1–306.

7. Sonuga-Barke EJS, Barton J, Daley D, Hutchings J, Maishman T, Raftery J, et al. A comparison of the clinical effectiveness and cost of specialised individually delivered parent training for preschool attention-deficit/hyperactivity disorder and a generic, group-based programme: a multi-centre, randomised controlled trial of the New Forest Parenting Programme versus Incredible Years. Eur Child Adolesc Psychiatry. 2018;27:797–809.

8. Wu Q, Li J, Parrott S, López-López JA, Davies SR, Caldwell DM, et al. Cost-Effectiveness of Different Formats for Delivery of Cognitive Behavioral Therapy for Depression: A Systematic Review Based Economic Model. Value in Health. 2020;23:1662–70.

9. Kyle SD, Siriwardena AN, Espie CA, Yang Y, Petrou S, Ogburn E, et al. Clinical and cost-effectiveness of nurse-delivered sleep restriction therapy for insomnia in primary care (HABIT): a pragmatic, superiority, open-label, randomised controlled trial. The Lancet. 2023;402:975–87.

10. Hollis C, Hall CL, Khan K, Novere ML, Marston L, Jones R, et al. Online remote behavioural intervention for tics in 9- to 17-year-olds: the ORBIT RCT with embedded process and economic evaluation. Health Technology Assessment. 2023;27:1–120.

11. Ondruskova T, Royston R, Absoud M, Ambler G, Qu C, Barnes J, et al. Clinical and cost-effectiveness of an adapted intervention for preschoolers with moderate to severe intellectual disabilities displaying behaviours that challenge: the EPICC-ID RCT. Health Technology Assessment. 2024;28:1–94.

12. Wright B, Kingsley E, Cooper C, Biggs K, Bursnall M, Wang H-I, et al. Play brick therapy to aid the social skills of children and young people with autism spectrum disorder: the I-SOCIALISE cluster RCT. Public Health Research. 2023;11:1–137.
